# Quantitative comparative analysis of amyloid PET images using three radiopharmaceuticals

**DOI:** 10.1007/s12149-023-01824-1

**Published:** 2023-02-07

**Authors:** Young Jin Jeong, Hyun Jin Yoon, Do-Young Kang, Kyung Won Park

**Affiliations:** 1 Department of Nuclear Medicine, Dong-A University Hospital, Dong-A University College of Medicine and Medical Center, 1, 3ga, Dongdaesin-dong, Seo-gu, Busan, 602-715 Republic of Korea; 2 Institute of Convergence Bio-Health, Dong-A University, Busan, Republic of Korea; 3 Department of Translational Biomedical Sciences, Dong-A University, Busan, Republic of Korea; 4 Department of Neurology, Dong-A University Hospital, Dong-A University College of Medicine, Busan, Republic of Korea

**Keywords:** Amyloid PET, F-18 florbetaben, F-18 flutemetamol, F-18 florapronol, Standardized uptake value ratio, Cut-off

## Abstract

**Objective:**

Amyloid positron emission tomography (PET) with F-18 florbetaben (FBB), F-18 flutemetamol (FMM), and F-18 florapronol (FPN) is being used clinically for the evaluation of dementia. These radiopharmaceuticals are commonly used to evaluate the accumulation of beta-amyloid plaques in the brain, but there are structural differences between them. We investigated whether there are any differences in the imaging characteristics.

**Methods:**

A total of 605 subjects were enrolled retrospectively in this study, including healthy subjects (HS) and patients with mild cognitive impairment or Alzheimer’s disease. Participants underwent amyloid PET imaging using one of the three radiopharmaceuticals. The PET images were analyzed visually and semi-quantitatively using a standardized uptake value ratio (SUVR). In addition, we calculated and compared the cut-off SUVR of the representative regions for each radiopharmaceutical that can distinguish between positive and negative scans.

**Results:**

In the negative images of the HS group, the contrast between the white matter and the gray matter was high in the FMM PET images, while striatal uptake was relatively higher in the FPN PET images. The SUVR showed significant differences across the radiopharmaceuticals in all areas except the temporal lobe, but the range of differences was relatively small. Accuracy levels for the global cut-off SUVR to discriminate between positive and negative images were highest in FMM PET, with a value of 0.989. FBB PET also showed a high value of 0.978, while FPN PET showed a relatively low value of 0.901.

**Conclusions:**

Negative amyloid PET images using the three radiopharmaceuticals showed visually and quantitatively similar imaging characteristics except in the striatum. Binary classification using the cut-off of the global cortex showed high accuracy overall, although there were some differences between the three PET images.

## Introduction

Amyloid positron emission tomography (PET) is widely used as an imaging biomarker for diagnosing cognitive impairment disorders such as Alzheimer’s disease. Currently, several radiopharmaceuticals for amyloid PET imaging are used clinically and for research, with F-18 florbetaben (FBB) [1], F-18 flutemetamol (FMM) [2], and F-18 florapronol (FPN) [3] approved for clinical use in Korea.

Both FBB and FMM have a high affinity for beta-amyloid plaques, and their clinical usefulness has been proven through histopathological confirmation. Since FBB is derived from Congo red [4] and FMM is derived from thioflavin T [5], the two substances differ structurally, which may lead to slight differences in kinetics and dynamics in the brain. In addition, because the distribution of the radioligands is different, there are considerable differences in the approved interpretation guideline [6]. For example, FMM PET requires an assessment of striatal binding, whereas FBB PET does not. However, despite these characteristic differences, several studies comparing FBB and FMM have reported a high interpretation concordance between the two radiopharmaceuticals [7–9].

FPN is a radiopharmaceutical used in amyloid PET imaging and is known as Alzavue^®^ in Korea (FutureChem; Seoul, Republic of Korea). Several studies comparing C-11 Pittsburgh compound B (PIB) and FPN have reported that the cortical uptake of FPN is similar to that of PIB [10–12], but there are no comparative studies with FBB or FMM. A high interpretation concordance and a high correlation of cortical uptake have been confirmed by previous studies comparing FMM and FBB with PIB, respectively [13–15]. Therefore, a significant correlation of imaging characteristics is expected between FPN and either FMM or FBB, but a direct comparative study between them is necessary.

In this study, we used retrospective clinical data to investigate the differences and characteristics of amyloid PET images obtained using the three radiopharmaceuticals mentioned above. First, the range of cerebral cortical uptake in normal subjects was evaluated semi-quantitatively. Second, cut-off values were obtained to distinguish between positive and negative scans in the cognitively impaired patient group, and the diagnostic accuracy of this value was also investigated.

## Materials and methods

### Patients

Altogether, 605 subjects comprising 97 healthy subjects (HS) and 508 patients with mild cognitive impairment (MCI) or Alzheimer’s disease (AD) who had received amyloid PET between August 2016 and October 2020 were enrolled retrospectively in this study. A clinical diagnosis was made by a dementia specialist based on the clinical guidelines. The inclusion criteria for the HS were aged 40 years or older, with a Mini-Mental State Examination (MMSE) score of more than 28 and a Clinical Dementia Rating (CDR) scale of 0. MCI was diagnosed according to Petersen’s criteria [16], while an AD diagnosis was made based on the National Institute on Aging-Alzheimer’s Association research criteria [17]. Anyone with insufficient clinical information, a structural brain disorder, or who moved their head significantly during PET acquisition was excluded.

Our Institutional Review Board reviewed and approved this retrospective study protocol (DAUHIRB-22-053). The requirement for informed consent was waived because this was a retrospective study using patients’ images. All methods were used in accordance with the relevant guidelines and regulations.

### Amyloid brain PET/CT acquisition

Participants underwent amyloid brain PET imaging using either FBB, FMM, or FPN. All PET examinations were performed with a Biograph mCT flow scanner (Siemens Healthcare GmbH, Germany). Injection dose and imaging times were varied according to the recommended protocol for each radiopharmaceutical proposed by the ligand manufacturers. FBB PET and FMM PET images were obtained 90 min after the intravenous injection of 300 MBq of FBB and 185 MBq of FMM. FPN PET images were obtained 30–60 min after the intravenous injection of 370 MBq of FPN. All PET scans were acquired for 20 min in the 3-dimensional (3D) and list mode from the skull vertex to the skull base. All PET images were reconstructed using the following parameters: 400 × 400 × 110 matrix with 2 × 2 × 2 mm voxel size using the 3D ordered-subsets expectation maximization algorithm (iterations = 2, subset = 21, time-of-flight and point-spread-function).

Unenhanced computed tomography (CT) scans were carried out using a 40-slice CT scanner before obtaining a PET image with an automatic exposure control technique using CARE Dose4D and CARE kV (Siemens Healthcare GmbH, Germany). CT images were reconstructed on a 500 × 500 × 110 matrix using an iterative reconstruction algorithm.

### Image analysis

#### Visual analysis

The PET images were interpreted visually by three nuclear medicine physicians with certification and experience in amyloid PET readings. Three physicians independently classified PET images into positive or negative scans according to the interpretation guidelines for each radiopharmaceutical. If there was a discrepancy in the classification, final decisions were made by consensus. The classification results determined under the agreement of the three physicians were considered the gold standard.

#### Quantitative PET image analysis

All PET images were analyzed semi-quantitatively using the syngo.MI Neurology on the syngo.via platform (Ver. VB20A, Siemens Healthcare GmbH, Germany) [18]. The standardized uptake value ratio (SUVR) was calculated using the “Ratio Analysis” function in the syngo.MI Neurology application. The Ratio Analysis function enables a comparison of one or more regions of interest in the patient brain to obtain ratios of the uptake in those regions. In the dedicated application, the PET volume is spatially normalized to a template built on Montreal Neurological Institute space, using the composite automated anatomical labeling atlas and a volume-of-interest (VOI) set. The representative areas were set up as the striatum, anterior and posterior cingulate, precuneus, frontal, parietal, temporal, and occipital lobes, and global brain by reconstructing the VOI of the atlas. We calculated the SUVRs of the representative areas and used the cerebellar cortex for reference. The global SUVR was calculated without the striatum since there was no consensus as to whether or not to include it.

We compared SUVRs to evaluate the normal distribution of each radiopharmaceutical in the cerebral cortex in three amyloid PET images in the HS group. The cut-off value of the representative regions for each radiopharmaceutical that can distinguish between positive and negative scans was obtained for all groups. Subsequently, the accuracy of each cut-off value was confirmed by consensus-based visual classification.

### Statistical analysis

We assessed the differences between group characteristics using one-way ANOVA and chi-squared tests. A one-way ANOVA was performed to compare SUVR values of the representative regions for each radiopharmaceutical in the HS group, with a post-hoc analysis performed if the ANOVA showed significant differences. The performance of the cut-off value obtained through the receiver operating characteristic (ROC) curve was confirmed through sensitivity, specificity, positive predictive value, accuracy, and the areas under the ROC curve (ROC-AUC). We performed the statistical analyses using the NCSS 2022 Statistical Software (NCSS, LLC. Kaysville, Utah, USA) and R v3.6.3 (Institute for Statistics and Mathematics, Vienna, Austria; www.R-project.org), with *p* < 0.05 denoting statistical significance.

## Results

### Patients and visual imaging characteristics

Table 1 shows the participants’ demographic information. Of the 605 subjects, 228 underwent FBB PET, 204 underwent FMM PET, and 173 underwent FPN PET. There were no significant differences in sex, age, MMSE score, or positive scan ratio of amyloid PET between the radiopharmaceuticals. However, a significant difference was shown in the disease proportion (*p* = 0.012) in the FPN PET group, where the proportion of HS was low, and the proportion of MCI patients was high.

**Table 1:** Participant demographics and clinical findings

Characteristics	FBB	FMM	FPN	*p*-value
Number of subjects	228	204	173	
Sex (M: F), female sex (%)	100:128, 56.1%	85:119, 58.3%	61:112, 64.7%	0.208
Age (years), mean ± SD (Range)	68.6 ± 9.6 (48–91)	71.2 ± 8.3 (49–92)	70.5 ± 7.3 (55–90)	0.096
MMSE, mean ± SD	20.7 ± 5.8	20.6 ± 5.2	21.3 ± 5.6	0.243
Diagnostic group (*n*, %)				0.012
Healthy	46 (20.2%)	34 (16.7%)	17 (9.8%)	
MCI	63 (27.6%)	59 (28.9%)	70 (40.5%)	
AD	119 (52.2%)	111 (54.4%)	86 (49.7%)	
Positive scan (*n*, %)	115, 50.4%	107, 52.5%	85, 49.1%	0.189

**Fig. 1:**
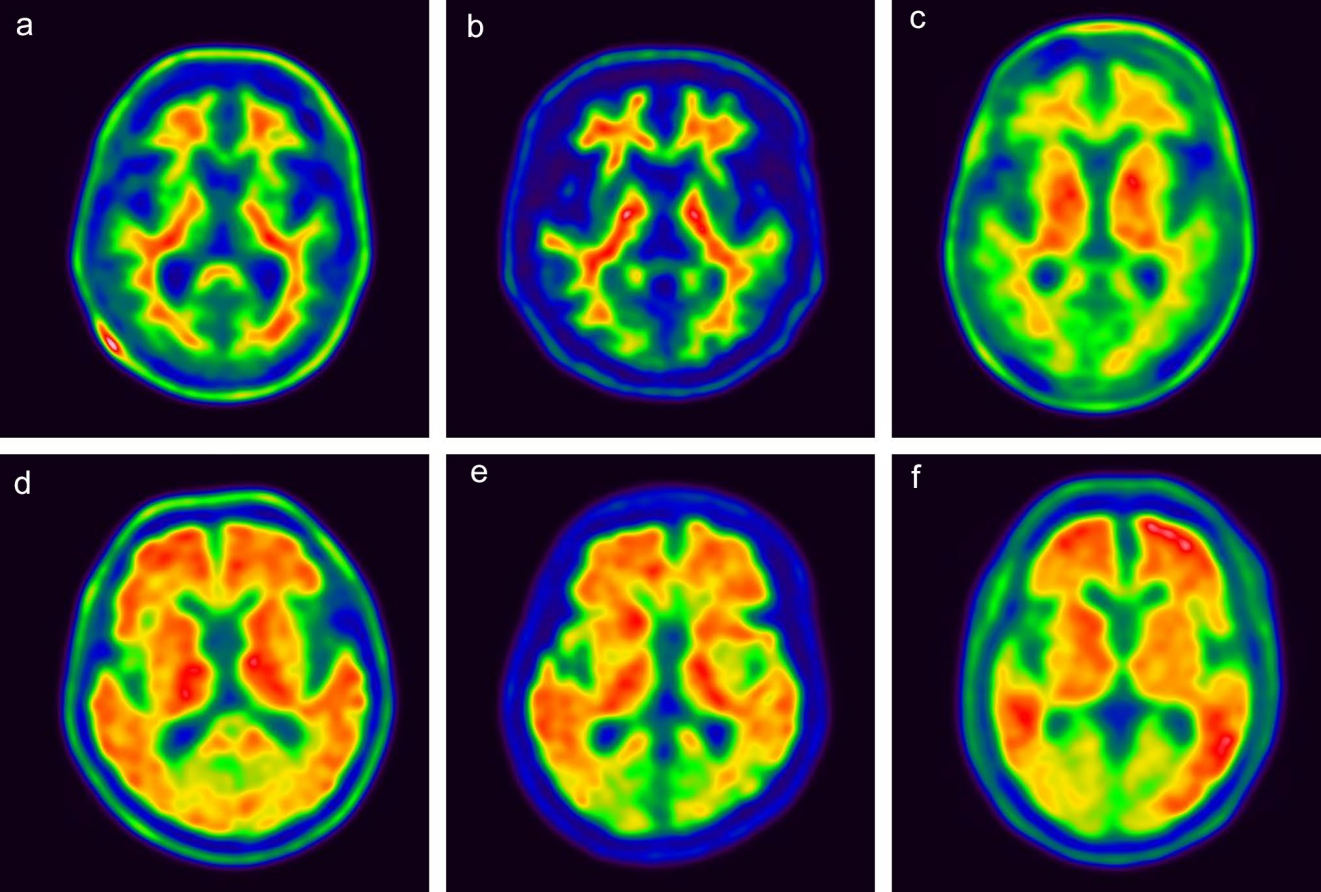
Representative amyloid PET images for each of the three radiopharmaceuticals (**a**, **d**: FBB, **b**, **e**: FMM, and **c**, **d**: FPN). Upper-row images are negative scans, and lower-row images are positive scans

In the amyloid PET images using the three radiopharmaceuticals, our study showed a clear difference between positive and negative images, and relatively similar findings were shown in each of the positive and negative images (Fig. 1). The characteristic findings were showing slight differences between the PET images. For example, the contrast between the white matter or amyloid deposit area and the background was high in the FMM PET image (Fig. 1b, e), while the striatal uptake was higher in the negative scan of the FPN PET image than in the other negative images (Fig. 1c). Although FBB PET images should be displayed in grayscale according to the manufacturer’s recommendation, the same rainbow color scale was applied in this study for easy comparison between the three radiopharmaceuticals.

### Regional SUVR in healthy subjects

**Fig. 2:**
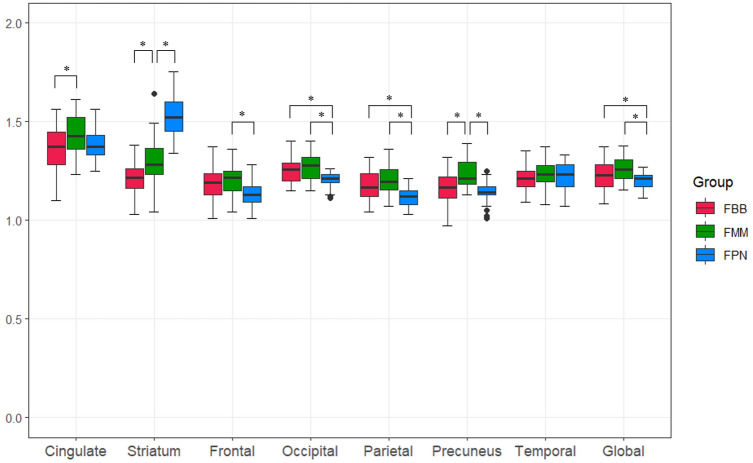
Comparison of cortical SUVRs in the HS group

In the SUVR comparison of representative regions in the HS group, significant differences were shown in all regions except for the temporal lobe, and there was no consistency in such differences (Table 2). Regardless of statistical significance, SUVR in all regions except the striatum was highest in FMM PET. In the frontal, parietal and occipital lobes, FPN PET had a significantly lower SUVR. In the precuneus, FMM PET showed the highest SUVR at a significant level. The SUVR in FMM was also significantly higher compared with FBB PET in the cingulate gyrus. In the global cortex, the SUVR was significantly greater in FMM PET (1.26 ± 0.06) than in both FBB PET (1.23 ± 0.07) and FPN PET (1.20 ± 0.05). The striatum showed a slightly different pattern from other areas. In the striatum, the SUVR of three PET images showed a significant difference, and the SUVR of FPN PET showed the highest value. These characteristic findings of the striatum were also plotted in a bar graph (Fig. 2).

**Table 2:** Summary statistics of cortical SUVR in the HS group

Regions	Radiopharmaceutical	SUVR (mean ± SD)	Range	*p*-value	Post hoc test
Cingulate	FBB	1.36 ± 0.12	1.10–1.56	0.007	FBB vs. FMM
	FMM	1.44 ± 0.11	1.23–1.61		
	FPN	1.38 ± 0.08	1.25–1.56		
Striatum	FBB	1.21 ± 0.08	1.03–1.38	< 0.001	FBB vs. FMM vs. FPN
	FMM	1.30 ± 0.12	1.04–1.64		
	FPN	1.52 ± 0.11	1.34–1.75		
Frontal	FBB	1.18 ± 0.08	1.01–1.37	0.009	FMM vs. FPN
	FMM	1.21 ± 0.07	1.04–1.36		
	FPN	1.14 ± 0.08	1.01–1.28		
Occipital	FBB	1.26 ± 0.06	1.15–1.40	0.002	FBB, FMM vs. FPN
	FMM	1.27 ± 0.07	1.15–1.40		
	FPN	1.20 ± 0.04	1.11–1.26		
Parietal	FBB	1.18 ± 0.07	1.04–1.32	< 0.001	FBB, FMM vs. FPN
	FMM	1.20 ± 0.07	1.07–1.36		
	FPN	1.11 ± 0.06	1.03–1.21		
Precuneus	FBB	1.17 ± 0.08	1.02–1.32	< 0.001	FBB, FPN vs. FMM
	FMM	1.23 ± 0.05	1.13–1.39		
	FPN	1.14 ± 0.07	1.01–1.25		
Temporal	FBB	1.21 ± 0.06	1.09–1.35	0.359	
	FMM	1.23 ± 0.06	1.08–1.37		
	FPN	1.22 ± 0.07	1.07–1.33		
Global	FBB	1.23 ± 0.07	1.08–1.37	0.002	FBB, FPN vs. FMM
	FMM	1.26 ± 0.06	1.15–1.38		
	FPN	1.20 ± 0.05	1.11–1.27

### Regional and global cut-off values in all subjects

For each radiopharmaceutical, we obtained the optimal cut-off SUVR for the representative regions that can distinguish between positive and negative scans using amyloid PET images for all the study groups (Table 3). Cut-off values showed similar patterns in most areas, and no area showed significantly higher accuracy than others. In general, FMM PET showed the highest value in all areas, while FPN PET had the lowest value except for the striatum and cingulate gyrus.

**Table 3:** Summary statistics of the ROC curve analysis in the entire subjects

Regions	Radiopharmaceutical	Cut-off value	Sens	Spec	PPV	Accuracy	ROC-AUC
Cingulate	FBB	1.33	0.904	0.991	0.991	0.947	0.979
	FMM	1.73	0.916	0.969	0.970	0.941	0.973
	FPN	1.44	0.812	0.671	0.704	0.740	0.815
Striatum	FBB	1.35	0.661	0.974	0.962	0.816	0.868
	FMM	1.49	0.860	0.907	0.911	0.882	0.933
	FPN	1.56	0.459	0.659	0.565	0.561	0.538
Frontal	FBB	1.29	0.878	0.965	0.962	0.921	0.958
	FMM	1.42	0.944	0.969	0.971	0.956	0.986
	FPN	1.24	0.718	0.83	0.803	0.775	0.837
Occipital	FBB	1.35	0.800	0.947	0.939	0.873	0.952
	FMM	1.47	0.869	0.979	0.979	0.922	0.960
	FPN	1.29	0.788	0.864	0.848	0.827	0.885
Parietal	FBB	1.35	0.887	1.000	1.000	0.943	0.976
	FMM	1.48	0.935	1.000	1.000	0.966	0.988
	FPN	1.22	0.824	0.830	0.824	0.827	0.898
Precuneus	FBB	1.33	0.904	0.991	0.991	0.947	0.979
	FMM	1.55	0.944	1.000	1.000	0.971	0.986
	FPN	1.26	0.859	0.784	0.794	0.821	0.887
Temporal	FBB	1.31	0.904	0.947	0.946	0.925	0.970
	FMM	1.44	0.916	1.000	1.000	0.956	0.987
	FPN	1.31	0.835	0.852	0.845	0.844	0.895
Global	FBB	1.35	0.922	0.991	0.991	0.956	0.978
	FMM	1.50	0.935	0.990	0.990	0.961	0.989
	FPN	1.29	0.906	0.761	0.786	0.832	0.901

*Sens* sensitivity, *Spec* specificity, *PPV* positive predictive value, *ROC-AUC* area under the receiver operating characteristic curve

In FBB and FMM PET, the sensitivity, specificity, positive predictive value, and accuracy of the cut-off values in most areas showed very high values overall. In particular, ROC-AUC showed values of 0.95 or higher except for the striatum. By contrast, FPN PET showed relatively low values, with ROC-AUC values less than 0.90 in all regions. In all PET images, the striatum showed lower accuracy than other regions.

**Fig. 3:**
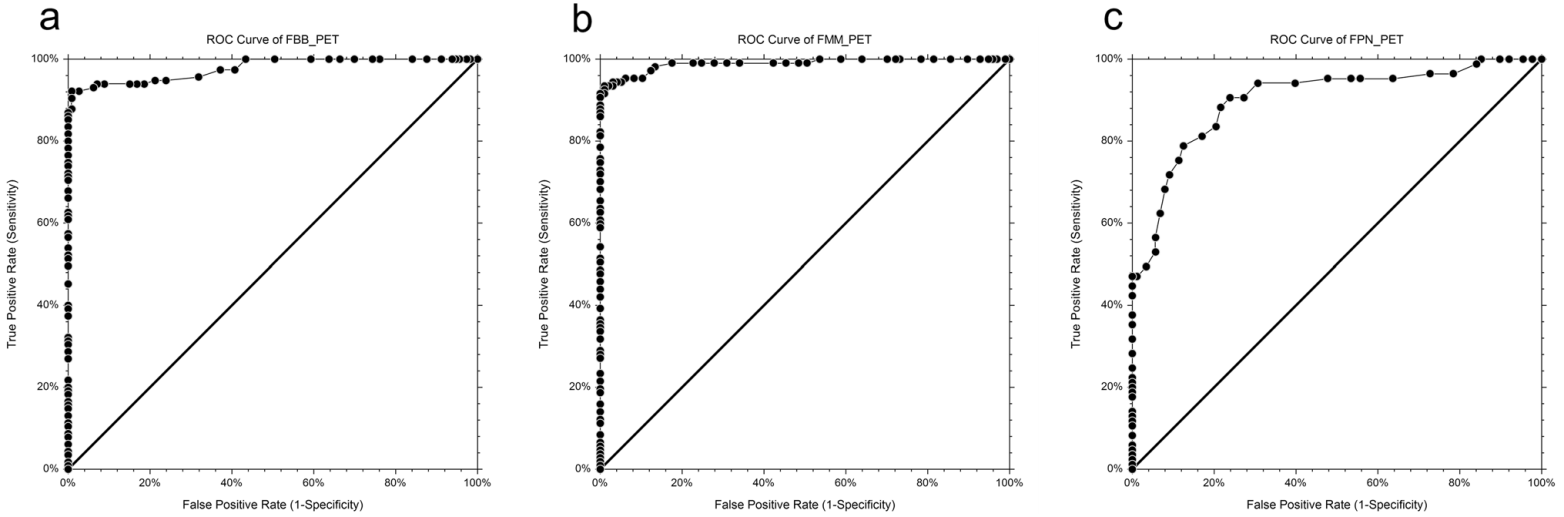
ROC curves for the global SUVR among amyloid PET images using FBB (**a**), FMM (**b**), and FPN (**c**)

The global cut-off values were 1.35, 1.50, and 1.29 for FBB PET, FMM PET, and FPN PET, respectively. The ROC-AUC for global cut-off value was highest in FMM PET at 0.989, although FBB PET also had a high value of 0.978, while FPN PET had a relatively low value of 0.901 (Fig. 3).

## Discussion

This study aimed to semi-quantitatively investigate the characteristics of amyloid PET images using FBB, FMM, and FPN. To accurately compare PET images using different radiopharmaceuticals, the study should be conducted with the same participants. However, it would be difficult to take three PET images for all subjects simultaneously. Although this study is a retrospective study using data for clinical practice, it is thought that a certain degree of tendency can be confirmed because the sample size is large (605) subjects.

When the study subjects’ characteristics were assessed, our study showed that there was a significant difference in the composition ratio of the subjects’ diseases by group according to the radiopharmaceutical. This is attributable to the nature of the retrospective study, whereby the subjects were randomly included. The rate of HS in FPN PET subjects was relatively low at 9.8%, while the rate of MCI was higher at 40.5%. Although there were differences in the proportion of diseases, this study aimed to determine amyloid positive or negative rather than to classify disease using amyloid PET. The positive rates of amyloid PET in all groups were 50.4% in FBB, 52.5% in FMM, and 49.7% in FPN, showing statistically similar rates. Therefore, it can be assumed that the difference in disease composition did not have a significant effect on diagnostic performance when distinguishing positive from negative results.

When the negative amyloid PET images of HS were visually compared, the basic image characteristics were similar between the three groups. White matter and gray matter were clearly differentiated in the negative images but merged and became indistinguishable in the positive images, showing common image findings. However, in the negative FMM PET image, the contrast between gray matter and white matter was relatively high compared with other PET images. This aspect was also confirmed in the quantitative analysis. In the comparison of negative images, we can infer that the SUVR of FMM PET was higher in all regions than other PET images due to the difference in contrast. Characteristically, striatal uptake in the negative FPN PET images was increased compared with other PET images visually. Regarding SUVR, FPN PET was 1.52 ± 0.11, which was relatively greater than FBB PET (1.21 ± 0.08) and FMM PET (1.30 ± 0.12). Beta-amyloid deposits in the striatum are associated with cognitive decline and are also associated with more advanced disease, so striatal findings are becoming an area of academic interest [19, 20]. However, whether or not the striatum is included in the evaluation area when visually interpreting the amyloid PET image depends on the radiopharmaceutical used. In the approved interpretation guideline, the striatum is not included in the evaluation area in FBB PET and FPN PET, although the guideline mentions that striatal uptake should be checked in FMM PET to ensure that the striatal gap is maintained [6]. In general, it is not clear why striatal uptake is seen in negative FPN PET images, unlike in negative FBB PET and FMM PET cases. However, these striatal characteristics should be understood when interpreting the FPN PET image.

In the negative images of the HS group, the SUVR of the representative regions was not statistically different in the temporal lobe, although the remaining areas showed significant differences. In all areas except the striatum, the SUVR was highest in FMM PET, which is thought to be due to the high lesion-to-background ratio, as mentioned above. Although there were some differences by area, SUVR decreased sequentially in most areas in FMM PET, FBB PET, and FPN PET, and the global SUVR showed the same trend. Regardless of statistical significance, the differences in SUVR between the three radiopharmaceuticals in each representative area were within 0.09, showing small differences of less than 8%. In a previous study comparing the SUVR of FBB PET and FMM PET in HS, the cortical composite SUVR obtained by setting the cerebellar cortex as the reference area was reported to be 1.13 ± 0.04 and 1.14 ± 0.05 in FBB PET and FMM PET [21]. This study showed a slightly lower SUVR compared with our study, which is likely caused by the difference in VOI settings in the representative region. The regional SUVR of FPN in HS could not be compared because there were no previous studies to compare it with.

Another purpose of this study was to compare diagnostic performance through a cut-off setting. We measured the cut-off value for each area that determines negative from positive for each radiopharmaceutical, with all areas showing similar findings to the negative scan results except in the striatum. The cut-off value was highest in FMM PET, followed by FBB PET and FPN PET. The ROC-AUC for these values were 0.946–0.979 for FBB PET and 0.933–0.989 for FMM PET, showing relatively similar ranges. By contrast, FPN PET showed a relatively high accuracy of 0.815–0.901 but lower values than the other two PET images. In the PET images using the same radiopharmaceutical, there were no significant differences in ROC-AUC in any of the regions except for the striatum. It takes 10–15 years from the onset of beta-amyloid deposition to the appearance of symptoms, and beta-amyloid deposits have reached saturation levels at the time of hospital admission [22]. For this reason, we can assume that the deposition of beta-amyloid by region is not significantly different in our study. In the striatum, the cut-off value in FPN PET was 1.56, which was higher than the other sites, while the ROC-AUC was low at 0.538. It is thought that FPN PET had a lower accuracy because negative scans also showed increased uptake in this area. Additionally, we compared striatal SUVR between HS and patient groups (MCI and AD) in three PET images. Striatal SUVR in HS and patient groups showed significant differences in FBB PET (1.20 ± 0.11 vs. 1.40 ± 0.24, *p* < 0.001) and FMM PET (1.30 ± 0.12 vs. 1.61 ± 0.32, *p* < 0.001), but FPN PET (1.52 ± 0.11 vs. 1.53 ± 0.19, *p* = 0.923) showed very similar values. In the case of FPN PET, when compared again by dividing into HS, MCI, and AD groups, each striatal SUVR was 1.52 ± 0.11, 1.53 ± 0.15, and 1.52 ± 0.17, showing no statistical difference (*p* = 0.684). The uptake of FPN in the striatum does not seem to correlate with disease progression. From this point of view, it is better not to include the striatum in the criteria for determining a positive or negative scan when interpreting the FPN PET image.

The global cut-off values and their ROC-AUC, which are the same concept as the composite cut-off value, were 1.35 and 0.978 in FBB, 1.50 and 0.989 in FMM, and 1.29 and 0.901 in FPN. Although the accuracy of FMM PET was the highest, it was similar to FBB PET. Although the accuracy of FPN was relatively low, it was still high at 0.901. Given this, the method of determining positive and negative using the cut-off value of the global area can also be assumed to be highly accurate. Previous studies have used various reference regions such as the pons, the whole cerebellum, the cerebellar cortex, or cerebral white matter to obtain cut-off values. Among them, we checked studies using the cerebellar cortex. In the case of FMM PET, a multicenter study reported an accuracy of 0.983 using a cut-off value of 1.57, showing a similar result to our study [23]. Sabri et al. showed a diagnostic accuracy of ROC-AUC of 0.914 (89% sensitivity and 92% specificity) with a threshold of 1.48 in an FBB PET study [1].

In this study, SUVR was used for quantitative comparison, but it cannot be used for direct comparison between different radiopharmaceuticals. Recently, the centiloid method was introduced to directly compare the cortical uptake of radiopharmaceuticals [24, 25]. This method for comparing FBB PET and FMM PET has been applied in several studies, but no study has applied it to FPN PET. In addition, since our study was a retrospective comparison of patient image data, we used SUVR. In the future, additional studies will be necessary to apply the centiloid method. To measure SUVR, it is necessary to set a reference region; the cerebellar cortex was used in this study. According to the interpretation guidelines, FBB PET uses the cerebellar cortex as the reference region, while FMM PET and FPN PET commonly use pons. Since this study aims to compare the characteristics of three radiopharmaceuticals, we were concerned that errors might occur because of the different reference regions. Therefore, the cerebellar cortex, which is generally known to be the last to deposit beta-amyloid, was used as the reference region for all three radiopharmaceuticals.

One limitation of this study is that it used retrospective data from a single hospital. For the measured quantitative value to be accepted as a universal result, image data from other environments, such as the Alzheimer’s Disease Neuroimaging Initiative, will be needed. Since this study only used data obtained from one hospital, we cannot extrapolate these findings more generally. However, since the amyloid PET imaging was performed using a consistent protocol using one PET/CT device in the same hospital, technical bias could be excluded depending on the device or acquisition method. Although the cut-off value presented in this study cannot be directly applied in other hospitals, we believe that the general characteristics of the three radiopharmaceuticals can be identified in the negative and positive images. In addition, while programs such as Statistical Parametric Mapping, PMOD, and freeSurfer are generally used during analysis to obtain the SUVR, this study used syngo.MI Neurology application, which takes automated SUVR measurements using CT-based spatial normalization techniques to analyze regional amyloid loading. Although syngo.MI Neurology is provided by PET/CT vendors, several studies have shown that there is no significant difference in results when using the program compared with other analysis programs. Since the measured values using syngo.MI Neurology in various studies have been reported as research results, the same method was applied in our study [26–29].

In conclusion, the amyloid PET images using the three radiopharmaceuticals showed visually similar imaging characteristics except in the striatum in the negative PET image. Although they show relatively similar SUVR values, they cannot be used for direct comparison by radiopharmaceuticals, so additional studies including studies using the centiloid method, are necessary. In addition, binary classification using the cut-off of the global cortex showed high accuracy overall, although there were some differences between the three PET images.

## Data Availability

The data that support the findings of this study are available from the corresponding author upon reasonable request.
